# Aesthetic experiences and their transformative power: a systematic review

**DOI:** 10.3389/fpsyg.2024.1328449

**Published:** 2024-10-03

**Authors:** Marta Pizzolante, Matthew Pelowski, Theresa Rahel Demmer, Sabrina Bartolotta, Eleonora Diletta Sarcinella, Andrea Gaggioli, Alice Chirico

**Affiliations:** ^1^Research Center in Communication Psychology (PSICOM), Catholic University of the Sacred Heart of Milan, Milan, Italy; ^2^Department of Cognition, Emotion, and Methods in Psychology, Faculty of Psychology, University of Vienna, Vienna, Austria; ^3^Vienna Cognitive Science Hub, University of Vienna, Vienna, Austria; ^4^IRCCS Istituto Auxologico Italiano, Milan, Italy

**Keywords:** transformative experiences, aesthetic, art, emotion, transformation

## Abstract

**Background:**

Transformative experiences (TEs) have been conceptualized in many ways, contexts, magnitudes, and durations, but at their heart, they entail some manner of adjustment, which contributes to changing individuals’ worldviews, actions, views of others and/or their own feelings, personality, and identity. Among the many elicitors identified as being able to foster TEs, an emerging body of literature has suggested that TEs might be prevalent in aesthetics or emerged from encounters with human art. Beyond denoting ordinary moments characterizing our daily lives, art and aesthetics could occasionally represent profound changes, causing shifts in our perceptions, beliefs and understanding of the world. However, in the realm of psychological inquiry, the extent to which art and aesthetics can be considered potential catalysts for transformation remains a topic of debate. Furthermore, a comprehensive identification of the key psychological components that contribute to the process of transformation before, during, and after aesthetic engagement is still missing.

**Aims:**

This systematic review endeavors to address these gaps by synthesizing literature on aesthetic transformative experiences either from the field of psychology or explicitly delving into the psychological impact of transformative experiences within the realm of art and aesthetics. It encompasses both theoretical and empirical papers to determine key aspects and psychological components that characterize TEs.

**Methods:**

Two major electronic databases were systematically searched. The review was conducted in accordance with Liberati et al. (2009) and PRISMA guidelines. All stages of the review were conducted independently by three researchers, and the protocol was published on PROSPERO (Registration no.: CRD42022298655).

**Results:**

Although 39.440 studies were identified, only 23 peer-reviewed articles were included in this review, as most studies did not explicitly delve into the long-lasting psychological impact of art and aesthetics.

**Discussion:**

The results confirm the potential of art and aesthetics as elicitors of transformation regardless of the type of artwork and the usage context. Moreover, it also identifies some psychological components necessary for transformation in the realm of art and aesthetics, including facilitating conditions/pre-expectations, cognitive discrepancy, epiphany and insight, and several after-effects on the recipient.

**Conclusion:**

The review aids in refining and enriching the concept of transformative experience, paving the way for further research and applications in various fields, including not only psychology but also education and therapeutic interventions.

**Systematic review registration:**

https://www.crd.york.ac.uk/prospero/, identifier CRD42022298655.

## Introduction

The concept of transformative experiences (TEs) has gained increasing attention across diverse domains and disciplines. This heightened interest has led to comprehensive exploration of these complex phenomena in various fields including education (Mezirow, 1997, 2018), anthropology (Van Gennep, 1908; Turner, 1974), technology (Gaggioli, 2016), philosophy (Paul, 2014; Carel and Kidd, 2020), neurobiology (Kielhöfer, 2011; Brouwer and Carhart-Harris, 2020) and religion (James, 1902; Stronge et al., 2021).

In the realm of psychology, empirical evidence has significantly contributed to the definition of TEs. These experiences have been deemed as extraordinary and unique events that bring about lasting and sometimes irreversible changes in individuals’ self-conception, worldviews, relationships, personality, and identity (Miller and C’de Baca, 2001). The characteristics of these experiences, which can take the form of an epiphany or a sudden insight, are reported to be remarkably consistent across cultures (Gaggioli, 2016). However, they have also been found to affect individuals profoundly in different ways (Tedeschi and Calhoun, 2004; Hayes et al., 2007) at different stages of life (Mezirow, 1997), and, most importantly, in response to different elicitors (Chirico et al., 2022; Gaggioli, 2016).

Actually, TEs encompass religious conversions, self-transcendent, peak and mystical experiences, out-of-body and near-death experiences, and psychedelic journeys, as elucidated and organized in a recent conceptual analysis of TEs by Chirico et al. (2022).

Although all of these experiences, with their respective elicitors, of our engagements with the environment, the supernatural, or with each other, and the unique transformative potential that seems to be contained within them and our human psychology itself, are compelling in their own right. One particularly salient topic that has emerged in recent years involves transformations brought about by art and aesthetics (e.g., see Pelowski and Akiba, 2011; Pelowski et al., 2017; for earlier reviews on the theme, see Lasher et al., 1983; Carroll, 1988). That is, beyond an important aspect of more ordinary moments across our daily lives—of which they are certainly a key component (Saito, 2001)— emerging research has suggested that aesthetic experiences might transcend mere entertainment or sensory stimulation, profoundly affecting our perceptions, feelings, beliefs, and understanding of ourselves and the world around us (Leder et al., 2012; Pelowski and Akiba, 2011; Marković, 2012; Pelowski et al., 2017). For instance, delving into an immersive book, which has the power to profoundly engage and captivate the reader, can be likened to a “portal” to alternate dimensions: it submerges us in narratives and immerses us in the inner lives of characters (Braun and Cupchik, 2001). The power of storytelling offers new perspectives, challenges preconceived notions, and creates empathy for a wide range of human experiences (Meretoja and Davis, 2017). Dipping one’s Madeleine into tea and taking a taste—to take the classic example from Proust (1981)—may flood us with memories, but also new realizations about the past of our own life and relationships (see e.g., Adorno, 1973). Similarly, visiting an art exhibition provides an opportunity to encounter visually compelling and thought-provoking works, inviting us to question established beliefs and consider alternative viewpoints (Arnold et al., 2014). The contemplation of art stimulates our imagination, triggers reflection, and elicits complex emotional responses (Freedberg, 2013; Perlovsky, 2014).

Philosophy, particularly the philosophy of art, has long been concerned with understanding how art fosters processes of transformation on both individual and collective levels. Art is not confined to the representation of reality; it functions as a significant agent of cognitive, emotional, and ethical change. As Dewey (1934) argued, aesthetic experience is an active and immersive process, wherein the observer engages with the artwork in a way that reconfigures their perception of reality. This interaction with art facilitates a form of experiential learning, enabling individuals to reassess their beliefs and values. Gadamer (1986) further developed this idea by suggesting that art serves as a medium through which truth is disclosed, involving the observer in a dialogical process. In this engagement, individuals are not passive recipients but active participants in the creation of meaning, which can lead to transformative experiences that deepen their understanding of historical and cultural contexts. This concept aligns with Gadamer’s notion of “hermeneutic openness,” where interpreting art becomes a pathway to personal and communal growth.

More contemporary philosophers, such as Shusterman (2000), have expanded on these ideas by exploring the pragmatic dimensions of aesthetic experience. Shusterman emphasizes how art can enhance both intellectual understanding and the quality of everyday life. His concept of “somaesthetics” highlights the embodied nature of aesthetic experience, arguing that the transformative potential of art is closely tied to the physical and sensory aspects of human experience.

In the realm of psychology, only in recent times, several contributions have undertaken empirical investigations into the transformative potential of aesthetic experiences (Pelowski and Akiba, 2011; Pelowski et al., 2017). Furthermore, outside the realm of psychology, an expanding body of research, spanning fields from education to design, has also started to explore the potential of art and aesthetics as elicitors driving personal and societal transformations.

Nevertheless, due to the varied and diverse nature of the psychological literature on art/aesthetics, questions persist about to what extent art and aesthetics can be categorized as elicitors of transformation. For example, there is still a lack of synthesis regarding the variety of artworks (including traditional forms and new media), usage contexts, and methods for evaluating and comparing the transformative potential of art and aesthetics. Moreover, there is currently a lack of a complete understanding of the psychological components that define aesthetic transformative experiences.

The subsequent analysis serves as a first endeavor to address these knowledge gaps and offer a comprehensible and relevant synopsis of the current body of literature pertaining to the transformative power of art. Our contribution is situated within the psychological literature on transformative experiences (TEs). Specifically, we aim to structure a discussion regarding the potential inclusion of art and aesthetics as elicitors of such TEs in order to establish a fundamental basis for subsequent empirical investigations, thereby enhancing our understanding of the transformative potential of aesthetic experiences.

## Research questions

This systematic review explores the extent to which art and aesthetics can be considered elicitors of personal transformation regardless of the type of artwork, context, and methodological approach used to assess their transformative potential. Additionally, the review elucidates the key psychological components contributing to the transformative process during and after aesthetic experience.

To do this, the current work synthesizes all the existing contributions in the psychological literature on the topic as well as studies explicitly addressing the psychological components of TEs within the domain of art and aesthetics.

This systematic review is guided by two primary research questions:

RQ1: To what extent can art and aesthetics be classified as transformative elicitors, and how does this categorization vary across different types of artworks, usage contexts, and the methodological approaches employed to evaluate their transformative potential?

RQ2: What are the fundamental psychological components characterizing aesthetic transformative experiences?

To deepen our understanding of the concepts of “aesthetic experience” and “transformation” and to guide our study selection, we draw upon recent theoretical advancements in both areas.

Within the psychological literature, the concept of aesthetic experience is highly relevant yet often poorly specified. It is frequently referred to using various terms such as aesthetic “engagement” (Brinck, 2018; Williams et al., 2023), “encounter” (Csikszentmihalyi and Robinson, 1990), “appreciation” (Leder and Nadal, 2014), “perception” (Biaggio and Supplee, 1983) or “engagement” and “encounter” with art (Shim et al., 2019; Csikszentmihalyi and Robinson, 1990), together with “perception” and “appreciation” of art (Crozier and Chapman, 1984; Roald, 2008). These terms are commonly used to describe the process by which individuals interact with and respond to art and aesthetics. In this review, we also use these to denote the broader concept of aesthetic experience.

Also, we refer to the definition of aesthetic experience provided by Mastandrea et al. (2019) and Marković (2012), which state that aesthetic experiences involve the appreciation of aesthetic objects, resulting in a particular kind of “pleasure”. This pleasure is distinct because it is not derived from the utilitarian properties of the objects but is intrinsically linked to their inherent aesthetic qualities. Thus, aesthetic experiences can emerge from the appreciation of human-made artifacts, such as artworks, including (but not limited to) poetry, sculpture, music, and visual arts. They can also arise from natural objects that possess aesthetic value, such as sunsets or mountain vistas (Mastandrea et al., 2019).

In this review, we focus on aesthetic experiences specifically associated with the appreciation of artworks, and we exclude those experiences that involve the production or creation of art.

With respect to the concept of transformation, according to Chirico et al. (2022), TEs are: “brief experiences perceived as extraordinary and unique, leading to enduring and potentially irreversible outcomes that change individuals’ self-conception, worldviews, perceptions of others, as well as their personality and identity” (p. 14). Within this framework, two core components emerge: (1) epistemic expansion and (2) emotional complexity. Additionally, the authors identified two relevant psychological factors: *facilitating conditions* and *recipient aftereffects.* The former pertains to particular conditions, such as dispositional traits and contextual factors, which consistently serve as recurring triggers for various TEs. The latter category relates to specific aftereffects, which have the potential to influence individuals’ cognition, emotions, and personality over an extended period. Their distinction has long been a fundamental criterion for defining TEs (Chirico et al., 2022).

In the context of aesthetics and art, as proposed by Pelowski and Akiba (2011) and in the more contemporary model of art perception outlined by Pelowski et al. (2017), transformation takes place when individuals perceive a misalignment between their pre-existing cognitive frameworks, expectations, or schemas, and the stimuli presented within artworks. Resolving this cognitive dissonance−often triggered by ambiguity or complexity in interpreting symbolic elements−leads to an “epiphany”−a sudden realization or insight. This profound understanding emerges as a result of the cognitive dissonance resolution provoked by the aesthetic encounter. Importantly, this cognitive dissonance comes along with significant affective responses. These emotional reactions play a crucial role in the transformative process, as the dissonance not only challenges cognitive structures but also evokes strong emotional experiences. These affective responses can range from initial confusion or frustration to feelings of e.g. satisfaction, joy, or even awe, upon resolving the dissonance.

The transformative process comprises four major stages and is influenced by five contextual factors. Each stage – pre-encounter, cognitive mastery, secondary control, meta-cognitive assessment, and immediate outcomes – corresponds to specific psychological factors, including physiological responses, cognitive activity, and affective states.

In summary, Chirico et al.’s (2022) conceptual analysis underscores two fundamental psychological components inherent to TEs: facilitating conditions and aftereffects, which encompass the enduring influence of the experience on the individual. Furthermore, we have incorporated insights from the theoretical models put forth by Pelowski and Akiba (2011) and Pelowski et al. (2017) to identify supplementary psychological factors linked to cognitive, emotional, and physiological responses to experiences categorized as transformative, as well as the ultimate outcomes of these experiences. The two models have been included together as they offer distinct yet complementary contributions. While the former establishes the foundation for empirical analyses of transformative experience in a cross-disciplinary and cross-domain context, the latter focuses specifically on the domain of aesthetics.

## Methods

### Protocol and registration

This systematic review was registered on the pre-registration platform PROSPERO # CRD42022298655. To conduct this systematic review, we followed guidelines proposed by Liberati et al. (2009). This involved five steps:

a) Initial identification of objectives and goals of the study;
b) A systematic search, in compliance with the eligibility criteria previously identified;
c) An assessment of the validity of the findings (here we used the Downs and Black 26-item QAT scale developed by Downs and Black, 1998);
d) A systematic synthesis of the findings;
e) A final discussion of the findings.

### Identifying relevant studies

A systematic search of the literature was performed in two academic databases: PubMed and Scopus. Google Scholar was also used as an additional academic search engine to ensure that the maximum number of relevant psychological contributions were retrieved. The search was focused on aesthetic experiences with the aim of being transformative, according to the definition and factors identified by the most recent integrated interdisciplinary conceptualization on TEs proposed by Chirico et al. (2022) and recent models describing aesthetic transformative experiences (Pelowski and Akiba, 2011; Pelowski et al., 2017).

We sought articles from any time until August 2023, the end of this search. We utilized the retrieval of relevant articles with the following search terms:

(“Aesthetic experience*” OR “Aesthetic engagement” OR “Aesthetic perception” OR “Aesthetic appreciation” OR “Consuming art” OR “Producing art” OR “Art*” OR “Aesthetic*” OR “Artistic product*” OR “Aesthetic product*”) AND (“Transformation” OR “Transformative” OR “Change” OR “Outcome*” OR “Emotion*” OR “Cognition” OR “Emotional response* OR “Cognitive response*” OR “Physiological response*”).

The first part of the search index concerns terms and words related to the experience of art and the fruition of aesthetic stimuli. The second part includes terms and words related to the dimension of transformation in terms of a long-lasting impact. This search string was applied to the title, abstract, full-text, and author keywords. Google Scholar was used for backward reference searching to run general searches of specific references and to identify relevant articles.

### Study selection

Peer-reviewed articles – original studies as well as theoretical papers or reviews - with the following characteristics, published from the beginning of the literature until August 2023, were included:

– Written in English or translated into English;
– Dealing with stimuli classified by the authors as “art” or, more generally, involving aesthetic experiences;
– Explicitly addressing the transformative side of said experiences in terms of a detectable psychological impact, according to the definition given, and psychological factors identified by the recent conceptual analysis by Chirico et al. (2022) and models accounting for transformation in the realm of art and aesthetics (Pelowski and Akiba, 2011, Pelowski et al., 2017).

Blog entries and websites - even though they may offer valuable insights and be maintained by reputable scholarly organizations - were omitted from consideration due to the challenges associated with comparing them to other types of literature.

The selection of our inclusion criteria for this systematic review requires two fundamental considerations. First, in pursuit of a comprehensive analysis of TEs within the realm of art and aesthetics, we opted not to restrict our selection solely to studies within the psychological literature. This choice was made because numerous studies outside the psychological field explicitly address and provide in-depth and quantifiable insights into the psychological impact of transformative aesthetic experiences.

Secondly, we aimed to encompass both review articles, such as literature reviews, systematic reviews, and meta-analyses, as well as empirical research articles. We deliberately included theoretical studies in our review due to their prevalence in discussions concerning the psychological effects of art and aesthetic experiences. Excluding theoretical papers would not have reflected the totality of current discussion and data in this entirety, and thus would have been potentially detrimental to the comprehensiveness of our analysis. These theoretical contributions significantly contribute to shaping our understanding and conceptualization of transformative aesthetic experiences, providing valuable insights and perspectives that complement empirical research.

Consequently, articles with the following characteristics were excluded:

– Dealing with art and aesthetic experiences but not explicitly assessing their psychological impact in terms of durable and/or irreversible change;
– Addressing transformative experiences but related to a different, unrelated (e.g., non-art/aesthetic) topic;
– Involving clinical populations (i.e., participants affected by psychological, neurological, and/or cognitive disorders).

Excluding articles involving clinical populations allows us to focus specifically on studies related to aesthetic experiences in non-clinical contexts. By doing so, we aim to gain a clearer understanding of the transformative potential of art and aesthetics for the general population without the potential confounding effects introduced by clinical conditions. This approach helps maintain the scope of our review and ensures a more coherent analysis of the research pertinent to our research question.

The initial search elicited 38,440 articles from the two databases and 176 from the reference review, which were retrieved with Google Scholar. 3,239 duplicates were identified and removed, leaving 35,201 articles to be screened. The initial screening of studies was based on their abstracts and titles, excluding noticeably irrelevant studies based on the inclusion/exclusion criteria priorly listed.

**FIGURE 1:**
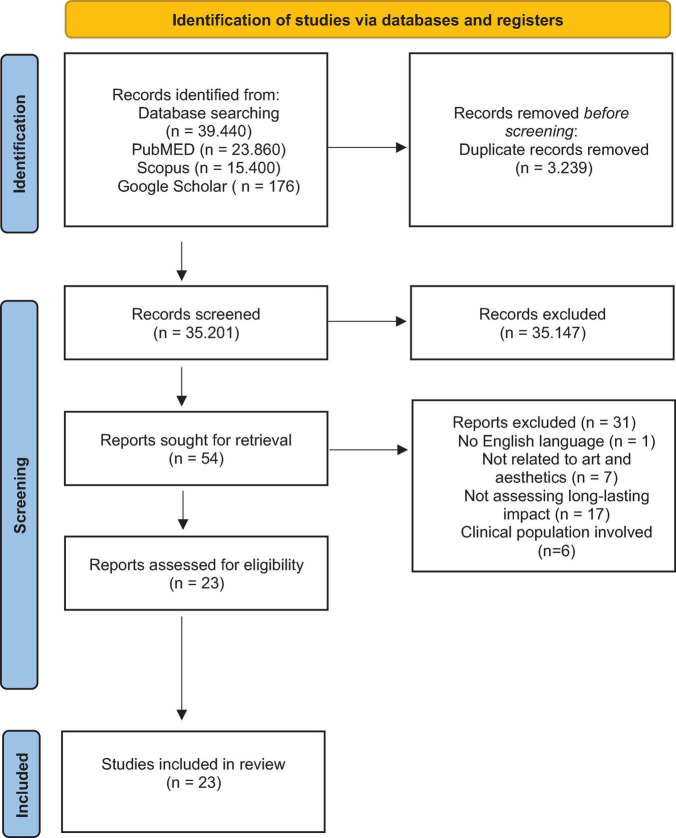
Flowchart of the included and excluded articles from the assessment of screening and eligibility process.

This large number of records was retrieved due to the wide initial search criteria that were adopted to avoid missing potentially relevant articles. However, following our above refinement criteria, in total, 54 articles were identified as appropriate for inclusion, and moved to the second screening round. The second round of screening was based on the full text of the articles, and the first (M.P.) and fourth (S.B.) authors independently reviewed each using the inclusion/exclusion criteria set before the search, as suggested by Levac et al. (2010). The second author (M.P.) and third author (T.D.) adjudicated when there were disagreements about whether to include the data extracted. In total, 23 articles were identified as appropriate for inclusion and relevant to the current review. Out of these, 12 were theoretical in nature, with the remaining 11 being empirical studies. The authors reviewed all articles independently. All reviewers together conjointly shaped the categories and themes of the review based on the data extraction process. A PRISMA flow diagram that depicts the flow of information through the different phases of the articles and maps out the number of papers identified, included, and excluded, and the reason for exclusions is shown in Figure 1.

### Risk of bias assessment

To assess the risk of bias and the overall methodological quality of the studies included in our analysis, we employed the Downs and Black 26-item Quality Assessment Tool (QAT) scale (Downs and Black, 1998). Our decision to employ this rigorous assessment tool was influenced by its well-established validity and reliability within the domain of systematic reviews (Deeks et al., 2003). The scale offers a comprehensive evaluation of study quality on a 26-item checklist, encompassing aspects such as reporting, external quality, internal validity bias, and internal validity confounding. Each item is scored either 0 or 1, with one exception in which the score range of 0–2 is applicable, resulting in a maximum possible score of 27. Scores can be interpreted as “excellent” (24–28 points), “good” (19–23 points), “fair” (14–18 points), or “poor” ( < 14 points), providing a standardized measure of study quality.

As the scale is primarily designed to evaluate the methodological rigor and quality of empirical studies – which is particularly relevant when assessing potential sources of bias in data collection, analysis, and reporting – we opted to utilize it exclusively for empirical research papers within our systematic review.

### Data extraction

A spreadsheet software (i.e., Microsoft Excel) was used to collect and catalog the articles. A copy of the sheet was made available also on an open access software (i.e., Google Docs).^1^

All articles found by searching for the above keywords have been cataloged according to the following scheme:

– title;
– author(s) and year of publication;
– type of study;
– study design;
– number of participants;
– type of art/aesthetic experience;
– field/domain of application;
– methods/instruments;
– presence/absence of a control condition;
– facilitating conditions;
– physiological responses;
– affective responses;
– cognitive responses;
– final outcome;
– specific effects on the recipient (*aftereffects*).

### Reporting the results

The findings of this systematic review are presented to address the two research questions.

To report the findings related to the first research question (RQ1), we have organized the studies by providing a comprehensive overview of both theoretical (e.g., study type) and empirical studies (e.g., participant count, study design). Additionally, we describe the types of art or aesthetic experiences considered and the various contexts in which they are applied. A summary table has been created to synthesize these findings, ensuring clarity and ease of reference (see Table 1).

**TABLE 1:** Detailed summary of the considered studies (i.e., type of study, field/domain of application, study-design, number of participants, methods/instruments used, comparison) (alphabetical order).

Title	References	Type of study	Type of artwork	Field/domain of application	Study-design	Number of participants	Method/Instrument used to detect transformation	Comparison
The Restorative and Transformative Power of the Arts in Conflict Resolution	Bang, 2016	Review	Visual arts, Music, Dance	Social	N.A.	N.A.	N.A.	
ART FOR CHANGE: Transformative learning and youth empowerment in a changing climate	Bentz and O’Brien, 2019	Research article	Visual arts, Storytelling	Social/Education	Uncontrolled before and after design	24	Survey, Group discussion	N.A.
Transformative Learning in the Art Museum: A Methods Review	Chisolm et al., 2020	Review	Visual arts	Education	N.A.	N.A.	N.A.	N.A.
Patterns of receptive and creative cultural activities and their association with perceived health, anxiety, depression and satisfaction with life among adults: the HUNT study, Norway	Cuypers et al., 2012	Research report	Visual arts, Poetry, Music, Dance, Theater	Social	Population-based study	50.797	Survey	N.A.
What is literature for? The role of transformative reading	Fialho, 2019	Review	Literature	N.A.	N.A.	N.A.	N.A.	N.A.
Art, Emotion, and Existential Well-Being	Funch, 2021	Review	Visual arts, Music, Movies	N.A.	N.A.	N.A.	N.A.	N.A.
Reflection, refraction, resilience: the transformative potential of art	Gaetani et al., 2021	Reflection	Music, visual arts	Education			Thematic groups, workshops	N.A.
Teaching and Learning Science for Transformative, Aesthetic Experience	Girod et al., 2010	Research article	Visual arts	Education	Quasi-experimental	N.A.	Self-report measures, Interviews	A traditional teaching method based on cognitive, rational framework
Cutting Deep: The Transformative Power of Art in the Anatomy Lab	Grogan and Ferguson, 2018	Research article	Visual arts	Education	Action research	N.A.	Interviews, Reflective Writing	N.A.
Art projects as transformative services to integrate refugees	Gross et al., 2021	Research article	Visual arts, Music	Social	Mixed-method approach	770	Survey, Semi-structured interviews	N.A.
Synchronization, Attention and Transformation: Multidimensional Exploration of the Aesthetic Experience of Contemporary Dance Spectators	Joufflineau et al., 2018	Research article	Dance performance	Art performance	Controlled before and after design	12	Two cognitive tasks, self-report measures	A dance performance not based on contemplative practice
Effects of Literature on Empathy and Self-Reflection: A Theoretical-Empirical Framework	Koopman and Hakemulder, 2015	Review	Literature	N.A.	N.A.	N.A.	N.A.	N.A.
Designing and Evaluation of an Artistic Experience for the Development of Empathic Capacity: “Stepping into Others’ Shoes”.	Martínez-López de Castro et al., 2022	Research article	Visual arts	Education	Ethnographic research	71	Reflective Writing, Focus groups	N.A.
Transformative Learning and the Arts: A Literature Review	Blackburn Miller, 2020	Review	Visual arts, Poetry, Storytelling, Movies	Education	N.A.	N.A.	N.A.	N.A.
Experience design and the dimensions of transformative festival experiences	Neuhofer et al., 2020	Research article	Visual arts, Music, Dance	Art performance	Constructivist research design	31	Semi-structured interviews	N.A.
Transnational Refugees: The Transformative Role of Art?	O’Neill, 2008	Review	Visual arts, Poetry	Social	N.A.	N.A.	Interviews, Reflective Writing	N.A.
5x5x5 = Creativity: Art as a Transformative Practice	Paris and Hay, 2020	Research article	Visual arts, Poetry	Education	Action research	N.A.	Open interviews, Reflective Journals	N.A.
Tears and transformation: feeling like crying as an indicator of insightful or “aesthetic” experience with art.	Pelowski, 2015	Research article	Visual arts	Art exhibition	Uncontrolled before and after design	79	Self-report measures	N.A.
Transformative art: art as means for long-term neurocognitive change.	Preminger, 2012	Hypothesis and theory article	Visual arts, Theater, Music, Video-games	N.A.	N.A.	N.A.	N.A.	N.A.
What Is Art Good For? The Socio-Epistemic Value of Art	Sherman and Morrissey, 2017	Review	Visual arts, Poetry, Music	N.A.	N.A.	N.A.	N.A.	N.A.
Empathy and the aesthetic: Why does art still move us?	Stamatopoulou, 2018	Review	Visual arts	N.A.	N.A.	N.A.	N.A.	N.A.
Aesthetic experience models human learning	Starr, 2023	Hypothesis and theory article	Visual arts, Poetry, Music	Human Learning	N.A.	N.A.	N.A.	N.A.
Transformative experiences at art museums to support flourishing in medicine.	Tackett et al., 2023	Research article	Visual arts	Education	Uncontrolled before and after design	5	4-week course with different activities (Visual Thinking Strategies, Back-to-back sketching, Group Poems, Jazz Seminar, Reflective Writing). Reflective writings, Interviews and Self-report measures were used to evaluate the intervention.	N.A.

To address the results for the second research question (RQ2), which aims at identifying the psychological components contributing to TEs fostered by art and aesthetics, we rely on the definition and criteria for TEs as outlined in the recent analysis by Chirico et al. (2022) and on theoretical models on transformation within the realm of aesthetic encounters (Pelowski and Akiba, 2011; Pelowski et al., 2017). These criteria are broadly categorized into facilitating conditions, physiological responses, cognitive responses, affective responses, as well as related final outcomes and aftereffects and re. To avoid redundancy with the discussion part, we have included a detailed table presenting the principal findings related to RQ2 (see Table 2). The comprehensive presentation of these findings are elaborated upon in the discussion section.

**TABLE 2:** Classification of the considered studies according to facilitating conditions, physiological, affective cognitive responses, final outcomes(s) and after-effects (alphabetical order).

Title	References	Facilitating conditions	Physiological responses	Affective responses	Cognitive responses	Final Outcome	After-effects
The Restorative and Transformative Power of the Arts in Conflict Resolution	Bang, 2016	Design of the experience following the principles of artist-audience synchronization	N.A.	Empathetic concern, Anxiety Reduction	Critical Reflection, Engagement, Meta-cognitive alertness	Innovation, Reflection	Long-lasting learning, Adoption of new perspective, Cooperative Relationship, Social Bounding
ART FOR CHANGE: Transformative learning and youth empowerment in a changing climate	Bentz and O’Brien, 2019	Creation of dialogic space	N.A.	Hope, Responsibility, Care, Solidarity, Serendipity, Uncertainty	Focus of attention on the self, Cognitive Dissonance, Complex dilemma	Creative Insight	Critical Thinking ability, Awareness, Sense of Empowerment, New Understanding, Values/Knowledge Reorientation
Transformative Learning in the Art Museum: A Methods Review	Chisolm et al., 2020	New and creative environment, Social Interaction	N.A.	Surprise, Fulfillment	Introspection, Critical Reflection, Cognitive challenge	Insight	Critical Thinking ability, Mindset Growth, Transformational Learning
Patterns of receptive and creative cultural activities and their association with perceived health, anxiety, depression and satisfaction with life among adults: the HUNT study, Norway	Cuypers et al., 2012	N.A.	N.A.	N.A.	N.A.	N.A.	Growth, new meaning, perceived health and well-being
What is literature for? The role of transformative reading	Fialho, 2019	N.A.	N.A.	Pleasure, Amusement, Engagement, Empathetic concern, Sympathy, Compassion	Flow, Reconceptualization, Perspective-taking	Insight into the Self/Into the Others	New sense of self, Modification of Personal Meaning
Art, Emotion, and Existential Well-Being	Funch, 2021	Aesthetic sensitivity/ Disposition	N.A.	Intense and overwhelming feelings, Pleasure, Tension, Anxiety and Fascination	Deep reasoning, Heightened Awareness, Stream of Thoughts	Aesthetic catharsis, Sense of wholeness, Being-moved	Existential well-being, Emotional Stability, Life satisfaction
Reflection, refraction, resilience: the transformative potential of art	Gaetani et al., 2021	Supportive environment, openness to art and reflection	N.A.	N.A.	Reflection, Insight, Self-awareness	N.A.	New behaviors, new knowledge, understanding attitudes, endeavors, deeper appreciation of life, resilience
Teaching and Learning Science for Transformative, Aesthetic Experience	Girod et al., 2010	N.A.	N.A.	Satisfaction	Cognitive dissonance, Higher interest	Aesthetic appreciation	Self-change, Broadened Perspective, Shift of Attitude, Long-lasting Learning
Cutting Deep: The Transformative Power of Art in the Anatomy Lab	Grogan and Ferguson, 2018	N.A.	N.A.	Relief, Satisfaction	Self-Reflection, Observation, Attentional Focus	Epiphany, Cathartic Effect	Long-lasting learning, Enhanced Resilience
Art projects as transformative services to integrate refugees	Gross et al., 2021	Openness, Social interaction	N.A.	Empathetic concern, Uncertainty, Social and Positive Emotions	N.A.	Creative Insight	New understanding/Meaning, Acceptance of Diversity, Social Bounding
Synchronization, Attention and Transformation: Multidimensional Exploration of the Aesthetic Experience of Contemporary Dance Spectators	Joufflineau et al., 2018	Design of the experience following the principles of dance-spectator synchronization	Slower breathing rate, spontaneous motor-tempo, general arousal	Feelings of fascination, boredom	More attention and vigilance, Higher absorption, direction of the attention and meta-cognitive attention	Artwork-spectator synchronization, Interpersonal resonance, Liking the performance correlated with the slowing down of the Breathing Rate	N.A.
Effects of Literature on Empathy and Self-Reflection: A Theoretical-Empirical Framework	Koopman and Hakemulder, 2015	N.A.	N.A.	N.A.	Focus on the present moment, Reflection	Insight, New Meaning, Epistemic Expansion	Empathy, Self-Reflection
Designing and Evaluation of an Artistic Experience for the Development of Empathic Capacity: “Stepping into Others’ Shoes”.	Martínez-López de Castro et al., 2022	N.A.	N.A.	Satisfaction, Surprise, Concern	Self-Reflection, Disinhibition	New meaning, Breaking Boundaries	Meaning construction, Emphatic Skills, Improved Social skills, Creativity and Self-Esteem
Transformative Learning and the Arts: A Literature Review	Blackburn Miller, 2020	In person facilitators	N.A.	Appreciation, Positive emotions	Imagination, disorienting dilemma, Reflection	Extra-rational knowledge, Insight	Long-lasting learning, Self/Others Awareness, Creativity skills, Empathy skills, Connection, Purposeful Chance
Experience design and the dimensions of transformative festival experiences	Neuhofer et al., 2020	Temporal and spatial liminality	N.A.	Positive emotions (awe, happiness, fulfillment)	Engagement, Flow	Epiphany, Quantum change, epistemic expansion	Cultural Identity, Sense of Community, Spiritual Growth, Meaning making
Transnational Refugees: The Transformative Role of Art?	O’Neill, 2008	Commitment, Potential and Dialogic Space	N.A.	N.A.	Awareness, Focus on the present moment, Reflection	Insight, Mutual Recognition	New knowledge, New understanding, Personal identity
5x5x5 = Creativity: Art as a Transformative Practice	Paris and Hay, 2020	Dialogic Space	N.A.	N.A.	Imagination, Focus on the self	N.A.	Creativity Skills, Intellectual development, Emotional Development, Social development
Tears and transformation: feeling like crying as an indicator of insightful or “aesthetic” experience with art.	Pelowski, 2015	Pre-expectations	Feel like crying	Tension, Confusion, Relief, Happiness	Cognitive discrepancy	Insight, Meaning/Understanding, Epiphany	Schema/Self-change
Transformative art: art as means for long-term neurocognitive change.	Preminger, 2012	N.A.	N.A.	Sense of unity, wholeness, awe	Use of imagery, imagination	N.A.	Neuro-cognitive changes: enhanced brain plasticity, enhanced brain activity in parahippocampal area, changes in cognitive functions
What Is Art Good For? The Socio-Epistemic Value of Art	Sherman and Morrissey, 2017	N.A.	Chills, tears, arousal	Negative, positive, mixed emotions	Memory integration, Directed attention, Meta-cognitive awareness	Aesthetic decision, evaluation; Insight and/or epiphany, Attention directed on the self	Self-understanding (e.g., belief/schema revision), Pro-social effects (e.g., developing empathy, perspective-taking, “practice” mentalizing), Well-being/flourishing/health, Perceptual skills (e.g., visual discrimination) Cognitive skills (e.g., creativity)
Empathy and the aesthetic: Why does art still move us?	Stamatopoulou, 2018	Disposition to empathy, Value Systems and Morality	N.A.	Emotional appraisal, Both positive and negative affect, Commotion	Reflexive Awareness, Focal attention, Focus on self- Flow	Aesthetic evaluation, Re-centering of the self	Empathic Skills
Aesthetic experience models human learning	Starr, 2023	Subjective tastes, Social Interaction, Vivid Perception, Pre-expectations	N.A.	Aesthetic Pleasure, Awe, Sublime	Enhanced Imagery, Mind-wandering	Successful identification of the percept (adherent to prior expectations), Peak-shift phenomenon, Insight	Enhanced learning capacity, Creative inspiration, Disinhibition
Transformative experiences at art museums to support flourishing in medicine.	Tackett et al., 2023	Creation of an unfamiliar context	N.A.	Wonder, awe	Quiet reflection, perspective-taking	Insight, Ambiguity tolerance	Human flourishing in terms of personal and professional growth, Renewed sense of purpose, Honesty, Re-connection with inner self and others,

## Results

Out of the 23 articles identified during the systematic review process, 12 were theoretical, including reviews and theory articles, while the remaining 11 were empirical research articles. One of the original research articles encompassed three distinct studies, resulting in a total of 13 individual empirical studies documented.

Subsequently, we assessed the quality of these 13 empirical studies using the QAT scale (Downs and Black, 1998).

Upon analyzing the 13 transformative experiences investigated in these studies, we calculated an average overall quality index on the QAT scale of 16.16 (SD = 2.40), with scores ranging from 13 to 21 (highest possible QAT = 27). These scores indicate that the studies fall within the range of fair to good validity and reliability with only one study performing poor.

### Description of included studies

Regarding the theoretical studies included in our systematic literature review, nine of them were literature reviews, two were hypothesis articles and one was a reflection article.

Empirical studies identified for this systematic review encompassed data from 51,789 participants in total, with sample sizes ranging from 5 to 50,797 participants. All included studies varied in terms of their study design. Studies included action research, a quasi-experimental design, mixed-methods, population-based designs, controlled before and after design and uncontrolled before and after design. Only two studies made comparisons with an active control group. The remaining studies did not utilize control groups.

### Description of type of art/aesthetic experience

Both theoretical and empirical articles included in our systematic review encompassed a diverse range of art forms and aesthetic experiences. It’s important to note that many studies included multiple types of aesthetic experiences within their analyses, allowing for a comprehensive exploration of the transformative potential across various art forms. They included:

• Visual Arts: The majority of the studies (83.3%) explored aesthetic experiences related to visual arts, such as paintings, sculptures, and art installations.
• Dance: Around one sixth (16.6%) delved into the transformative potential of dance performances, analyzing how movement and choreography influenced participants.
• Music: Around a third (33.3%) examined the power of music as an aesthetic experience, investigating how different compositions could impact individuals’ perceptions and emotions.
• Literature: One fourth (25%) focused on the aesthetic experience of reading and analyzing literary works, including novels, poetry, and essays.
• Video Games: One study explored the immersive experiences offered by video games, examining their potential for transformation and emotional engagement.

### Description of domain of application/usage context

We identified a wide range of domains and fields of application in which transformative aesthetic experiences were examined. These included:

• Artistic context: Three studies (13%) explored the transformative potential of aesthetic experiences within live art performances, such as theater, dance performances, and other staged events. One study focused on art exhibitions and galleries, investigating how curated visual displays could lead to transformative experiences for viewers.
• Educational context: A significant body of research (30%) analyzed the role of aesthetic experiences in education, considering how art and aesthetics could enhance learning and personal development.
• Social context: Several studies (22%) explored the potential of aesthetic experiences related to their potential to influence social attitudes, empathy, and community engagement.

### Psychological factors underlying aesthetic transformative experiences

#### Facilitating conditions

In our systematic literature review, we identified both participant-related dispositional traits and contextual design elements as facilitating conditions for transformative aesthetic experiences. Key dispositional factors included empathy, aesthetic sensitivity and openness, that is the willingness to embrace novel and diverse experiences. Pre-existing expectations (i.e., preconceived notions or anticipations about the aesthetic experience, such as expecting a profound emotional impact) and subjective preferences (i.e., individual tastes or inclinations towards certain art forms or styles) also influenced transformative outcomes.

Contextual design elements refer to the specific aspects of an environment or setting that are deliberately crafted to enhance the experience and engagement of participants (i.e., the intentional use of lighting, soundscapes, or spatial arrangements to evoke a particular emotional or cognitive response). These elements encompass the establishment of a dialogic space (i.e., a setting that encourages open and reflective dialogue among participants), temporal and spatial liminality (i.e., the creation of a ‘threshold’ experience where ordinary boundaries of time and space are suspended), and innovative environments (i.e., spaces that challenge conventional perceptions and expectations). Additionally, Joufflineau et al. (2018) found that unfamiliar and uncomfortable settings (i.e., environments that provoke a sense of disorientation or challenge one’s comfort zone) are particularly effective in fostering such transformation.

#### Physiological, cognitive, and affective responses

Transformative aesthetic experiences were linked to various physiological responses, including chills, tears, and arousal. Participants exhibited slower breathing rates, suggesting a calming effect, and one study by Pelowski (2015) reported sensations of wanting to cry, highlighting the emotional depth and intensity of these encounters.

Cognitive responses to transformative aesthetic experiences included heightened attention and vigilance, deep engagement or “flow”, and cognitive dissonance. Moments of self-reflection and introspection were common, along with an increased focus on others and a sense of disinhibition, reported by a study by Martínez-López de Castro et al. (2022).

Affective responses were diverse, encompassing positive emotions such as happiness, satisfaction, and surprise, as well as fascination, wonder, and awe. Initial feelings of boredom or confusion were often followed by relief and relaxation. Affective responses also included empathetic concern and feelings of care and responsibility towards others.

#### Final outcomes

The final outcomes of transformative aesthetic experiences, which occur immediately after the experience, varied widely. Again, in the study by Joufflineau et al. (2018), participants reported a deep sense of connection and interpersonal resonance, moments of epiphany and cathartic release, and an increased tolerance for ambiguity. These encounters often led to new meanings and the challenging of established norms, accompanied by epistemic expansion and creative insight.

#### After-effects

Unlike final outcomes, which are immediate, aftereffects refer to the long-term, enduring changes that persist over time. The aftereffects of transformative aesthetic experiences included lasting changes in self-concept and personal growth, enhanced empathy and social awareness, and long-lasting learning and critical thinking skills. These experiences also fostered emotional development, resilience, and creativity.

For a detailed presentation and explanations of these findings, please refer to the summary table provided below (Table 2). These concepts are further elaborated and specified in the discussion section.

## Discussion

This systematic review was conducted with two primary objectives. Firstly, we aimed to assess the extent to which art and aesthetics could be classified as elicitors of transformation, regardless of the type of artwork, the context in which they occur, or the methodological approach used to assess their transformative impact (RQ1). Secondly, we endeavored to identify the core psychological components that define transformative aesthetic experiences (RQ2). Our discussion has been structured to comprehensively address both research questions.

To achieve these objectives, we conducted an extensive review of the existing literature, encompassing studies from psychological literature as well as studies explicitly dedicated to understanding the psychological dimensions of TEs fostered by art and aesthetics. This comprehensive approach allowed us to extract valuable insights, providing a more profound understanding of the transformative potential inherent in aesthetic encounters and the fundamental psychological components that underpin them.

### Beyond beauty: are art and aesthetics elicitors of transformation?

In recent decades, there has been a notable shift in scientific inquiry that has expanded our understanding of the role of art and aesthetics in human experience. Historically, research within psychology and neuroscience primarily focused on unraveling the cognitive processes underpinning aesthetic judgments, individual preferences, and the neural mechanisms responsible for perceiving beauty. This narrow focus led to a limited perspective where art was often reduced to a matter of personal taste and subjective liking, largely sidelining its potential for profound impact (Sherman and Morrissey, 2017).

However, a significant shift occurred within psychological literature with the introduction of the concept of TEs. TEs are defined as experiences that bring about significant and lasting changes in individuals’ perceptions, emotions, beliefs, and sense of self (Chirico et al., 2022). Alongside this conceptual shift, several models identified art and aesthetic experiences as potential elicitors of transformation. These models provided a theoretical framework for exploring how art could serve as a catalyst for personal and societal change, ushering in a more comprehensive view of the transformative potential inherent in artistic encounters (Pelowski and Akiba, 2011; Pelowski et al., 2017).

The outcomes of this systematic review unequivocally confirm the transformative potential inherent in art and aesthetic experiences (RQ1). The comprehensive coverage of various art forms and domains within the selected studies, encompassing visual arts, dance, music, literature, and even video games, underscores the widespread occurrence of transformative encounters across diverse creative mediums. This inclusivity offers a multifaceted view of the transformative potential within art and aesthetics.

It is essential to acknowledge that a substantial portion of the studies in this review have predominantly focused on visual arts, such as paintings and sculptures. This emphasis on visual art may reflect a historical bias in prioritizing visual channels as a means of artistic expression and communication (Cherry, 2004; Qurbonova, 2021). Over the years, visual art has held a prominent place in the discourse surrounding aesthetic experiences and transformation, likely due to its long-standing recognition as a powerful medium for conveying and eliciting emotions (Panofsky and Drechsel, 1970; Silvia, 2005; Freedberg and Gallese, 2007; Leder et al., 2004; Menninghaus et al., 2015). Consequently, the wealth of literature in the realm of visual arts highlights a legacy of research that acknowledges its transformative capacities (O’Neill, 2008; Cuypers et al., 2012; Pelowski, 2015; Preminger, 2012; Bang, 2016; Girod et al., 2010; Sherman and Morrissey, 2017; Grogan and Ferguson, 2018; Bentz and O’Brien, 2019; Paris and Hay, 2020; Chisolm et al., 2020; Blackburn Miller, 2020; Neuhofer et al., 2020; Funch, 2021; Gross et al., 2021; Martínez-López de Castro et al., 2022; Gaetani et al., 2021; Stamatopoulou, 2018; Starr, 2023, Tackett et al., 2023).

The wealth of literature in visual arts, however, does not overshadow the transformative potential that other art forms possess. The studies examining dance, music, literature, and video games signify the expanding domains and the recognition of transformation in varied creative contexts. These findings suggest that art, across its myriad forms, wields the potential to act as a potent catalyst for personal and societal change, provided that researchers explore each medium with the same vigor and thoroughness (O’Neill, 2008; Cuypers et al., 2012; Koopman and Hakemulder, 2015; Preminger, 2012; Bang, 2016; Sherman and Morrissey, 2017; Joufflineau et al., 2018; Neuhofer et al., 2020; Blackburn Miller, 2020; Funch, 2021; Gross et al., 2021; Gaetani et al., 2021; Starr, 2023). The varied foci within these studies contribute to a broader understanding of transformation through art and aesthetics, encouraging a more inclusive perspective that embraces the full spectrum of human creativity.

This expansive range of art forms not only showcases the versatile nature of transformative aesthetic experiences but also underscores the potential applicability and relevance of these experiences in multiple domains and contexts. The diverse domains and fields of application in which transformative aesthetic experiences were examined reveal the multifaceted nature of these experiences. It is noteworthy that studies within the educational domain, that focuses on enhancing learning and personal development, have a well-established tradition, building upon the foundations laid by Mezirow (1997), Mezirow and Taylor (2009) and the concept of transformative learning. These studies reflect the enduring and robust interest in the educational sector in harnessing the potential of art and aesthetics for personal growth and knowledge acquisition (Girod et al., 2010; Grogan and Ferguson, 2018; Paris and Hay, 2020; Chisolm et al., 2020; Blackburn Miller, 2020; Martínez-López de Castro et al., 2022; Gaetani et al., 2021; Tackett et al., 2023).

Conversely, the expanding body of research conducted in a social context underscores a growing trend. These studies explore how transformative aesthetic experiences can convey significant messages, influence social attitudes, foster empathy, and promote community engagement (O’Neill, 2008; Cuypers et al., 2012; Bang, 2016; Bentz and O’Brien, 2019; Gross et al., 2021). The rising interest in social contexts signifies a realization of the persuasive power of art and aesthetics in addressing broader societal issues and inspiring collective change (Sidford, 2011; Glaveanu, 2017).

Furthermore, the various study designs found within the empirical studies identified in this systematic review contribute to a holistic comprehension of the transformative potential inherent in art and aesthetic experiences. However, it would becrucial to acknowledge the variations in design and the limited use of control groups in most studies. These factors have important implications for the strength of the evidence and our ability to draw robust conclusions.

The use of action research, quasi-experimental, mixed-method, population-based, and before-and-after designs reflects the multidimensional nature of transformative aesthetic experiences. These approaches allow researchers to capture both quantitative and qualitative aspects of transformation. The mixed-method studies, in particular, provide a richer understanding by combining numerical data with participants’ narratives and reflections. This comprehensive approach aligns with the complex and multifaceted nature of TEs.

### Pre-encounter with the arts: what are the conditions that facilitate aesthetic TE?

Our study results offer a perspective on the facilitating conditions leading to aesthetic transformation. These conditions encompassed both participant-related dispositional traits and elements linked to the design of the experiential context. Each condition is analyzed separately in the following.

The study of individual differences in art and aesthetics appreciation began with the application of psychology to education at the turn of the 20th century. These differences have often related to personality (Chamorro-Premuzic et al., 2009; Mastandrea et al., 2019; McManus and Furnham, 2006; Chirico et al., 2023), intelligence (Chamorro-Premuzic and Furnham, 2004; Furnham and Chamorro-Premuzic, 2004; Chirico et al., 2023), expertise (Belke et al., 2010; Silvia and Barona, 2009), and other personal traits.

In the current review, among dispositional factors, the importance of individual traits such as empathy, aesthetic sensitivity (Pelowski, 2015; Stamatopoulou, 2018), and openness (Gross et al., 2021; Funch, 2021) consistently emerged. Pre-existing expectations and subjective preferences were found to influence the likelihood of transformative outcomes (Pelowski, 2015; Starr, 2023).

As Stamatopoulou (2018) claims, empathy, as a personality trait, contributes to transformative outcomes by enhancing an individual’s ability to connect emotionally with the artwork and the experiences it portrays. This emotional connection can lead to profound personal insights and shifts in perspective, fostering greater self-awareness and understanding of others (Batson et al., 1997). Empathy enables individuals to experience the emotions conveyed through art, facilitating a deeper engagement that can potentially catalyze a TEs (Pelowski, 2015). Neuroscientific research on mirror neurons has provided additional insights into how empathy functions in the context of art (Piechowski-Jozwiak et al., 2017). Mirror neurons are a type of brain cells that respond both when an individual performs an action and when they observe the same action performed by another (Rizzolatti et al., 1999). These neurons are suggested to play a crucial role in the capacity for empathy, as they enable individuals to internally simulate and understand the experiences of others (Rizzolatti and Craighero, 2004). When engaging with art, mirror neurons may be activated as viewers perceive and emotionally resonate with the depicted actions and emotions, thus deepening their empathic response (Freedberg and Gallese, 2007). The connection between art and empathy has been explored extensively in psychological literature. Engaging with art can enhance empathic abilities by allowing individuals to vicariously experience diverse perspectives and emotions. This can lead to a greater understanding and appreciation of others’ experiences, fostering social cohesion and personal growth (Goldstein and Winner, 2012). Moreover, art can serve as a powerful tool for empathy training, helping individuals to develop greater emotional intelligence and compassion (Kaplan, 2020).

Another personality trait specific to the aesthetic context that emerged is aesthetic sensitivity. This trait is defined as the capacity to perceive and appreciate beauty and compositional excellence, and to evaluate artistic merit based on aesthetic standards (Corradi et al., 2020), also plays a crucial role. Funch (2021) argues that individuals with high aesthetic sensitivity are more likely to engage deeply with art, recognizing and valuing its elements, which can lead to significant personal reflections and transformative insights. This trait allows for a more immersive and impactful aesthetic experience, as individuals are attuned to the finer aspects of the artwork that may trigger profound emotional and cognitive responses. Additionally, openness, a personality trait characterized by creativity, imagination, and curiosity, is another key factor. This facet encompasses traits such as creativity, imagination, unconventionality, and curiosity, all sub-facets associated with a heightened inclination toward aesthetic experiences (Silvia et al., 2009). In one of the studies selected for the current review, Gross et al. (2021) measured the trait of openness and found it positively correlated not only with the willingness to engage in Integrative Art Projects (IAPs) but also with the transformative effectiveness of the intervention. IAPs are structured programs that combine various forms of artistic expression, such as visual arts, music, dance, and creative writing, to foster holistic personal development and self-expression.

This result is in line with previous literature showing that openness to experience is linked to a greater propensity for aesthetic appreciation and positive encounters with art (McCrae and Costa, 1985). This trait encourages individuals to explore new ideas and perspectives, making them more receptive to the potential for transformation through aesthetic experiences (Chamorro-Premuzic et al., 2007; Silvia et al., 2009). Individuals high in openness are more likely to seek out and engage with diverse and complex artworks, increasing the likelihood of experiencing transformative outcomes.

In addition to these traits, other factors such as cultural background, education level, and previous exposure to art may also play significant roles in shaping one’s capacity for aesthetic transformation. These elements, although not always explicitly mentioned in the reviewed studies, are hypothesized to influence the depth and nature of transformative experiences (TEs) (Pelowski et al., 2017). Moreover, these factors are deeply connected to another facilitating condition identified in this review: pre-expectations. According to two studies included in our review (Pelowski, 2015; Starr, 2023), pre-expectations are defined as the preconceived notions, attitudes, and anticipations that individuals bring to an aesthetic encounter. These pre-expectations can significantly shape the way an individual engages with and responds to art. Cultural background influences pre-expectations by providing a framework of values, traditions, and norms through which art is interpreted (Darda and Cross, 2022). For instance, individuals from cultures that highly value artistic expression may approach art with a greater sense of reverence and openness, which can facilitate deeper TEs.

Education level contributes to pre-expectations by equipping individuals with the critical skills and knowledge necessary to appreciate and analyze art. Those with higher levels of education, particularly in the arts, are likely to have more sophisticated frameworks for interpreting and engaging with artistic works, which can enhance the potential for transformative outcomes. Previous exposure to art also plays a crucial role in forming pre-expectations (Leder et al., 2014). Individuals who have been frequently exposed to art are likely to have developed a richer and more nuanced understanding of aesthetic experiences. This familiarity can make them more receptive to the emotional and cognitive impacts of art, thereby increasing the willingness to commit to the experience itself and the likelihood of the transformation.

Furthermore, our findings underscore the pivotal role of contextual elements in shaping transformative experiences. Key elements include the establishment of a dialogic space within the artistic or aesthetic encounter, the introduction of temporal and spatial liminality, and the creation of new and innovative environments. The establishment of a dialogic space involves creating an environment conducive to open dialogue and exchange between participants and the artwork. This space encourages viewers to engage in reflective conversations, fostering deeper connections and understanding (Paris and Hay, 2020; Blackburn Miller, 2020). For instance, in the study by Paris and Hay (2020), this space was created through a specific participatory program that engaged new audiences with contemporary arts, design, and creativity. This program included interactive workshops, guided discussions, and collaborative art-making sessions that allowed participants to share their interpretations and responses to the artworks.

By actively involving the audience in the creative process and encouraging them to voice their perspectives, the dialogic space breaks down barriers between the artist and the viewer, transforming the encounter from a passive reception to an active, co-creative experience. This engagement not only deepens the individual’s connection to the art but also facilitates mutual understanding among participants, as they are exposed to diverse viewpoints and interpretations.

Temporal liminality refers to creating an environment where participants feel detached from their usual sense of time. This can involve altering the flow of time within the experience, such as through prolonged engagement with a single piece of art or through immersive installations that blur the boundaries of time perception (Thomassen, 2015; Liedgren et al., 2023). Spatial liminality entails modifying physical elements to craft a unique and unfamiliar setting. As in the study by Neuhofer et al. (2020), this can be achieved by redesigning the physical space of the art venue, incorporating unexpected spatial configurations, in terms of visual aesthetics and design of the place, that stimulated the five senses and challenge participants’ usual spatial perceptions (Shortt, 2015).

Interestingly, one study even suggested that unfamiliar and uncomfortable contexts could foster transformative experiences. In the study by Tackett et al. (2023), which aims at supporting the flourishing of 3rd and 4th year medical students, these settings push participants out of their comfort zones, encouraging them to confront and reflect on their discomfort, leading to potential personal growth and transformation. A notable characteristic of these settings is their emphasis on simulated high-stakes scenarios. These scenarios are designed to mimic real-life medical emergencies and complex patient interactions, which are inherently stressful and demanding. By simulating these high-pressure environments, the study aims to prepare medical students for the realities of their future careers while simultaneously fostering resilience and adaptability.

In essence, both dispositional traits and contextual design play pivotal roles in the potential for aesthetic transformation. It is not an “either-or” scenario, but rather a synergy between individual disposition and contextual elements that can unlock the full potential of art and aesthetics as catalysts for personal and societal change. This insight underscores the importance of a holistic approach when leveraging art for educational or social purposes, recognizing that a combination of factors can lead to profound and meaningful transformation in the realm of aesthetics.

### During the aesthetic experience: the role of discomfort and disruption

Throughout history, artists have intentionally pushed boundaries, confronting audiences with uncomfortable truths, challenging preconceived notions, and disrupting established conventions (Walker, 1999). Similarly, viewers have often described being deeply disrupted and struck by their experiences with art.

As we explore the role of art in transformation, findings from our systematic review underlined the significance of cognitive discomfort as a powerful trigger for expanding perspectives and inciting change. Gadamer (1984) and Rothbaum et al. (1982) have asserted that this transition from discordance to self-schema transformation marks the distinction between basic perception to profound interpretation and personal development. In the realm of art, the ability to overcome discordance and achieve self-schema change serves as the demarcation line between a superficial or unfruitful interaction with art and an aesthetic experience characterized by novelty and profundity (Dufrenne, 1973; Adorno, 1984; Dewey, 2008).

While current modeling on art perception tends to focus exclusively on the moment of “aesthetic insight”, or peak of harmonious pleasure – when individuals are finally able to grasp the final meaning of an artwork and to resonate with it - the true transformative potential of art and aesthetics primarily emerges firstly from discomfort and disruption, which ultimately lead to self-schema transformation and to the creation of a new belief (Pelowski and Akiba, 2011; Pelowski et al., 2017).

Drawing from the insights of Pelowski and Akiba (2011) and the more recent VIMAP model proposed by Pelowski et al. (2017), it becomes apparent that the transformative potential originates from the observer’s pre-existing expectations regarding the artwork or the experience itself. The self-reflective process triggered when these expectations are disrupted marks the inception of metacognitive reevaluation, ultimately culminating in a modification of the self-schema.

Consider, for instance, an individual attending an avant-garde art exhibition with the preconceived expectation that art should conform to traditional forms and structures. Upon encountering a highly abstract and unconventional artwork that challenges these expectations, the individual experiences confusion and disorientation. This initiates a process of self-reflection as they contemplate why they find this artwork perplexing and why it does not align with their prior beliefs about art. Over time, this metacognitive revaluation may lead to a shift in their perception of art, making them more open to unconventional forms of artistic expression and, consequently, transforming their self-schema concerning art appreciation.

Also, in the context of transformative learning, as conceptualized by Mezirow (2003) and further elaborated by others, the concept of the “disorienting dilemma” holds a central place. This concept represents a critical juncture in the transformative process where individuals are confronted with a situation or information that fundamentally challenges and disrupts their pre-existing beliefs, assumptions, or expectations. It serves as a catalyst for inducing cognitive dissonance, a state characterized by the mental discomfort arising from holding conflicting or incongruent beliefs or attitudes.

The significance of discomfort and disruption as the initial stages of transformation is further validated by our findings. In his study, Pelowski (2015) accounts for the experience of feeling like crying as a physiological response to discomfort and consequent self-schema change. The author presents a dual-factor model proposing that tears result from a cognitive and physiological process initiated by an initial discrepancy in interaction with the artwork which led to feeling like crying and related emotional states (i.e., being moved). This is followed by a subsequent shift in schema or expectations, leading to resolution and, theoretically, a potential restructuring of the self.

In educational studies employing art and aesthetic experiences, students often encounter conflicting perspectives, especially when grappling with intricate subjects like climate change or complex scientific theories (Girod et al., 2010; Bentz and O’Brien, 2019). This cognitive dissonance materializes when students are presented with compelling evidence and arguments that challenge their previously held beliefs, attitudes, or existing knowledge on the topic. This dissonance typically manifests as feelings of confusion, discomfort, or even emotional tension (Girod et al., 2010).

To resolve this cognitive dissonance, individuals might embark on a process of self-reflection and discourse. If so, they engage during this phase in critical self-examination, questioning their existing beliefs while attempting to understand the reasons behind their discomfort. In this context, individuals may actively seek out additional information, participate in discussions with peers or educators, and critically assess the validity of various perspectives. Through this systematic process, they aim to reconcile the dissonance either by adapting and revising their existing beliefs or by incorporating new information into their worldview.

As we further explore, this experience of cognitive dissonance serves as a catalyst for self-reflection, the critical examination of assumptions, and a willingness to explore and embrace new viewpoints (Girod et al., 2010).

### Immediate outcomes of aesthetic transformation: insight and epiphany

Numerous immediate outcomes can be attributed to the experiences of cognitive dissonance and discrepancy that frequently manifest during aesthetic encounters. Central to these outcomes is the recurring theme of *insight* and *epiphany*, which can be defined as moments of sudden and profound understanding or realization.

The profound impact of art and aesthetic experiences on fostering such epiphanies has been extensively studied in the context of art therapy and art-based practices. In these settings, both the creation and appreciation of art and aesthetics have been linked to the analytical experience of insight, often described as “seeing in” (Rubin, 2011).

Furthermore, it is crucial to observe that the concept of “transformative learning,” which corresponds to the notion of sudden knowledge acquisition resulting from a profound cognitive reorganization, was initially introduced by Mezirow (1997, 2003) as a fundamental element of the Transformative Theory of Learning, primarily within the educational context. This theory consists of two fundamental elements: critical reflection or critical self-reflection, which involves individuals examining their underlying assumptions, and critical discourse, where learners validate their judgments and acquire new knowledge through discussions with other adults to explore further and refine their assumptions and realizations.

In the recent conceptual analysis conducted by Chirico et al. (2022), *epistemic expansion* emerged as one of the two phenomenological components of TEs. This concept refers to the acquisition of novel forms of knowledge, impacting both the self and the external world. To illustrate, within the context of spiritual experiences, *epistemic expansion* manifests as a profound sense of self-diminishment, accompanied by a deep feeling of interconnectedness with all living beings. During peak experiences, individuals perceive the world as inherently good, beautiful, and desirable, while simultaneously realizing the harmonious resolution of previously perceived polarities and dichotomies. Conversely, near-death experiences (NDEs) often entail the dissolution of bodily boundaries, an acute comprehension of the entirety of existence, and the sensation of standing at the precipice of an irreversible threshold. Additionally, those undergoing NDEs often encounter an altered subjective perception of time, which may expand or dilate, despite the entire experience lasting only a few moments. Furthermore, perceptions of space are frequently strained, distorted, and transcended, exemplified in cases of out-of-body experiences and NDEs.

In the studies included in this review, participants often articulate how aesthetic encounters initiate insights and cognitive shifts, motivating them to explore new meanings, challenge boundaries, and question prevailing ideologies. For instance, these encounters frequently prompt individuals to challenge established norms and beliefs concerning the self, others, and the world (Pelowski, 2015; Tackett et al., 2023). In a study by Neuhofer et al. (2020), attendees at transformative experiences festivals often reported a sense of accomplishment through the exploration of their inner selves, suggesting that this exploration can lead to the unlocking of existential questions and serve as catalyst for sudden realization about the world such as a benevolent and positive place.

In the study conducted by Bentz and O’Brien (2019), art was employed instrumentally to communicate and raise profound awareness about important social and environmental issues. Participants taking part in the designed project reported a sudden discovery about their “own consumerist interior”, indicating a newfound insight about themselves. Others stated that they immediately “changed behavior, which led to new routines, which then led to new thoughts,” or that their involvement in the project through experiential learning resulted in a “180-degree change.” Consequently, participants demonstrated increased awareness about issues related to water shortages, water quality, plastic pollution, and their ecological footprint.

As highlighted by the above findings, it’s important to note that encounters with art and aesthetics do not lead to the mere acquisition of knowledge but to a process by which participants construct meaning in their lives and develop a deeper understanding of themselves, others, and the world. This type of experience entails a profound shift in perceptual framework, prompting individuals to rapidly scrutinize their existing beliefs and perspectives critically. This examination enables them to adopt fresh viewpoints and accommodate novel insights and information.

Closely related to the concept of insight, another relevant outcome observed in some studies is the development of increased ambiguity tolerance among participants (Tackett et al., 2023). This suggests that transformative aesthetic experiences expand individuals’ capacity to embrace complexity and navigate emotional intricacies effectively. Such an outcome underscores the potential of aesthetics to enhance individuals’ ability to appreciate life’s nuances and navigate uncertainty.

As we explore in the next section, these epiphanies can have lasting effects on individuals, contributing to personal growth and heightened self-awareness and connection with others.

### Long-term aftereffects: personal growth, empathy and connection

The long-term aftereffects of transformative aesthetic experiences reveal a multifaceted spectrum of enduring personal and social growth, underscoring their profound and lasting impact on individuals. These aftereffects can be categorized across various domains, beginning with a significant shift in an individual’s schema and self-concept, as a consequence of the epistemic expansion process. This transformation results in a long-lasting alteration in how individuals perceive both themselves and the world around them, as demonstrated in studies by Pelowski (2015) and Girod et al. (2010). This transformation frequently extends beyond the individual, leading to personal and professional growth, characterized by a renewed sense of purpose, increased honesty, and improved self-esteem, as observed in research by Paris and Hay (2020), Neuhofer et al. (2020), and Tackett et al. (2023).

The aftereffects encompass interpersonal and social dimensions, as participants often report a reconnection with their inner selves and a heightened connection with others. This leads to improved empathic skills, enhanced social and cultural awareness, and a strengthened sense of community, as documented in studies by Cuypers et al. (2012), Paris and Hay (2020), Neuhofer et al. (2020), and Gross et al. (2021).

This is in line with previous literature in which art and aesthetic experiences have been recognized to have the unique ability to foster empathy and connection (Eisner, 2002; Goldstein and Winner, 2012). When we engage with art, whether through visual arts, literature, or performance, we often step into the shoes of others, experiencing the world from different viewpoints (Nussbaum, 1997). This empathetic connection has profound implications for social change: aesthetic encounters can lead to greater understanding and compassion for diverse perspectives, ultimately promoting tolerance and unity (Greene, 1995). In a world marked by division and conflict, the capacity of art to bridge gaps and generate empathy deserves attention and exploration (Zeki, 2001). These transformative encounters also foster long-term learning, honing critical thinking abilities and promoting the acceptance of diversity, thereby highlighting their profound educational and societal implications (Mezirow, 1991; Dewey, 1934).

Furthermore, transformative aesthetic experiences have a lasting impact on emotional development. They contribute to enhancing resilience, emotional intelligence, and creativity skills, as indicated by studies conducted by Bentz and O’Brien (2019).

## Conclusion

In this review, we undertook the systematic task of synthesizing the existing literature explicitly addressing the psychological impact and components underlying TEs in the domain of art and aesthetics.

Literature increasingly converges in considering art and aesthetics as potential elicitors of transformations (Pelowski and Akiba, 2011, Pelowski et al., 2017). This established understanding highlights how engaging with art, music, literature, or nature can evoke profound emotional responses, alter our mood, and also foster deeper individual psychological changes.

However, the literature lacks a comprehensive exploration of the nature and extent of these transformations. While it is evident that aesthetic experiences can challenge perspectives and provoke thought, the specific mechanisms and breadth of these changes remain under-researched.

In conclusion, building on a previous conceptual analysis of TEs by Chirico et al. (2022) and several theoretical frameworks by Pelowski and Akiba (2011) and Pelowski et al. (2017) that evaluated the temporal sequencing of aesthetic experiences (pre-encounter, during the experience, immediate outcomes, and long-term aftereffects), the current work achieved two main objectives related to RQ2 (What are the fundamental psychological components characterizing aesthetic transformative experiences?). First, it investigated the psychological dimensions of TEs identified by Chirico et al. (2022) within the context of aesthetic experiences. Second, it systematically examined how and when the effects of aesthetic transformation occur.

In the pre-encounter phase, we focused on the conditions that can predispose an individual to have a transformative aesthetic experience. During the aesthetic experience, we explored the role of discomfort and disruption as key elements that can catalyze change. Subsequently, we analyzed the immediate outcomes of aesthetic transformation, such as insight and epiphany, which represent moments of profound understanding and revelation. Finally, we considered the long-term effects, such as personal growth, empathy, and connection, highlighting the lasting impact of transformative aesthetic experiences.

This work not only helps to better operationalize the variables of interest in empirical experimental settings but also provides a useful framework for designing future studies. For instance, researchers and practitioners in the field can use this work to identify and measure specific pre-experimental conditions, critical experiential elements, and post-experiential outcomes, thereby facilitating a more precise design for aesthetic transformation assessment. Additionally, this work can help develop targeted interventions and educational programs that leverage the specific timing/ moment at which each psychological effect of aesthetic transformative occurs. For example, if an intervention aims to stimulate creativity and insight generation, it could be beneficial to design a task where participants first engage with aesthetic stimuli, followed by an insight generation task or assessment. Conversely, if the goal of another intervention is to strengthen the sense of belonging to a community, it might be more effective to focus on the longer period following the aesthetic experience and, therefore, longitudinal studies (Sherman and Morrissey, 2017).

Regarding RQ1, which concerns the potential to affirm the transformative nature of art and aesthetics regardless of the type of artwork, usage contexts, and the methodological approaches employed to evaluate their transformative potential, the conclusions we can draw correspond to the possible limitations of the present work. Primarily, these limitations pertain to the predominance of studies related to visual art compared to other art forms and usage contexts, as already mentioned in the discussions. This is likely due to the ease of designing studies with visual stimuli and the greater emphasis traditionally placed on visual art. This imbalance should be taken into consideration, as the prevalence of studies related to visual art skews results regarding the effects of aesthetic experiences, leading to less literature on music, dance, and other artistic forms.

Additionally, with respect to the methodological approaches, the aesthetic experiences under consideration often do not include control groups. In the present work, we found only two studies that included a control group. Although the absence of control groups does not invalidate the findings, it limits the ability to establish causal claims regarding the transformative effects of art and aesthetics. Control groups play a crucial role in distinguishing the impact of artistic or aesthetic experiences from other variables that might influence transformation (Skov and Nadal, 2023). This is particularly important when dealing with subjective and context-dependent phenomena like TEs.

To strengthen the evidence base in this field, future research should prioritize more rigorous study designs and more heterogeneous stimuli (visual, auditory, tactile etc.). Randomized controlled trials (RCTs) with well-defined control groups can help establish causal relationships and enhance the internal validity of findings. RCTs offer the advantage of experimental control, allowing researchers to manipulate variables related to art and aesthetics and assess their impact systematically (Lord et al., 2009; Mohr et al., 2009). While RCTs in the context of aesthetic experiences may pose practical and ethical challenges, they remain a gold standard for determining causality (Bolier et al., 2013; Weiss et al., 2016) and closing this gap should be a guiding principle for future empirical studies.

Nevertheless, this synthesis provides only a glimpse into the richness of this field as it stands at present. The thorough analysis of both theoretical and empirical studies has enabled us to assemble a mosaic of insights that shed light on the complex nature of transformative aesthetic experiences. Our investigation also highlights the need for continued research to delve deeper into the multi-layered complexity of these experiences.

For instance, the emergence of immersive technologies, including virtual and augmented reality, as innovative ways to encounter art and aesthetics, introduces a new layer of complexity and a new frontier for research and exploration (Candy and Edmonds, 2002; vom Lehn et al., 2007; Candy and Ferguson, 2014; Pizzolante and Chirico, 2022; Pizzolante et al., 2023, 2024). Furthermore, the introduction to these technologies lead to new questions about if and how they can sustain transformation or other kinds of psychological changes (Zubala et al., 2021; Neuhofer et al., 2020; Sherman and Morrissey, 2017). For example, can digital environments evoke transformative experiences similarly to traditional settings? What features of digital art or VR experiences contribute to their transformative potential? Are these transformations equivalent to those in traditional settings? Additionally, there is a need to investigate the long-term effects of technologically mediated experiences. Do they lead to sustained personal growth and social connectedness akin to traditional art forms?

These questions are critical, as more people, especially the youth, engage with these technologies. Understanding their potential can help create beneficial interventions leveraging their power.

The practical implications of this systematic review traverse multiple disciplinary boundaries, spanning the domains of psychology, education, therapy, and the arts. Acknowledging the profound potential of aesthetic experiences opens doors to enriching educational programs, therapeutic interventions, and the conception of artistic and cultural encounters. Educators, for instance, have long harnessed the transformative power of art and aesthetics to nurture critical thinking, empathy, and personal development among students (Peppler, 2010). Dewey, in his seminal work “Art as Experience” (1934), emphasized that aesthetic experience is integral to education. He argued that engagement with art fosters reflective thinking and emotional growth, encouraging individuals to perceive and interpret the world in novel ways. Through aesthetic experiences, students learn to appreciate complexity, embrace ambiguity, and develop a deeper understanding of themselves and others. Also, Mezirow’s theory of transformative learning also underscores the educational value of art and aesthetics. According to Mezirow (1991), transformative learning involves a profound shift in perspective, often triggered by a disorienting dilemma that challenges existing beliefs and assumptions. Art, with its capacity to provoke and disrupt, serves as an ideal catalyst for such transformative experiences. By confronting students with new perspectives and emotional challenges, aesthetic experiences can facilitate critical reflection and promote significant personal and intellectual growth. Thus, incorporating aesthetic elements into curricula provides students with unique opportunities to engage with the world profoundly. For example, through the study and creation of art, students can explore diverse cultural narratives, understand complex social issues, and develop a nuanced appreciation of human experiences. This approach not only enhances cognitive skills but also fosters empathy and emotional intelligence. Moreover, aesthetic education often extends beyond traditional classroom settings. Field trips to museums, art galleries, and theaters, as well as community-based art projects, provide experiential learning opportunities that reinforce classroom teachings and foster a lifelong appreciation for the arts.

In the realm of therapy, art and aesthetics can serve as powerful vehicles for emotional healing and profound personal growth. Therapists have the opportunity to explore the therapeutic utility of aesthetics, allowing individuals to express themselves in ways that may be difficult through conventional verbal communication.

For example, an individual struggling with trauma or emotional distress may find solace and healing through creating art or engaging with aesthetic experiences. The act of creating art, be it painting, sculpture, or even writing, can provide a safe and expressive outlet for exploring and confronting complex emotions (Geller, 2018). The aesthetics of the artwork itself can offer valuable insights into the individual’s emotional landscape, facilitating therapeutic breakthroughs (Tsiris, 2008; Sarasso et al., 2023).

Aesthetic experiences can also be applied in group therapy settings, where participants collectively engage in creating art or experiencing art forms. This shared aesthetic journey can foster a sense of community, empathy, and support, contributing to personal growth and resilience (Kapitan, 2011).

Moreover, the artistic community itself holds a pivotal role in fostering social transformation, advocating for empathy, and bridging gaps among diverse populations. Artists, curators, and cultural institutions can curate immersive environments and experiences that intentionally facilitate transformative encounters, paving the way for meaningful reflection and growth (Milbrandt, 2010; Clammer, 2014; Blackburn Miller, 2020).

In conclusion, by acknowledging and harnessing the transformative potential of aesthetics, we stand to cultivate a world characterized by deeper connections, heightened empathy, and enriched human experiences. Future research endeavors should embrace interdisciplinary approaches and innovative methodologies to further illuminate the processes and far-reaching consequences of transformative aesthetic encounters.

## Data Availability

The original contributions presented in the study are included in the article/supplementary material, further inquiries can be directed to the corresponding author.

## Footnotes

1
https://shorturl.at/cyUsm
